# Staging of suicidality in bipolar disorder: Findings from the FACE-BD cohort (FondaMental Advanced Centers of Expertise for Bipolar Disorders)

**DOI:** 10.1192/j.eurpsy.2025.10068

**Published:** 2025-07-22

**Authors:** Anna Maria Auxilia, Emilie Olié, Jonathan Dubois, Enrico Capuzzi, Valérie Aubin, Bruno Aouizerate, Frank Bellivier, Raoul Belzeaux, Caroline Dubertret, Dominique Januel, Emmanuel Haffen, Antoine Lefrere, Agnès Pelletier, Mircea Polosan, Roman Rey, Paul Roux, Ludovic Samalin, Raymund Schwan, Michel Walter, Antoine Yrondi, FondaMental Advanced Centers of Expertise in Bipolar Disorders (FACE-BD) Collaborators, Pierre Michel Llorca, Marion Leboyer, Bruno Etain, Philippe Courtet

**Affiliations:** 1 Department of Emergency Psychiatry and Acute Care, CHU Montpellier, IGF, Univ. Montpellier, CNRS, INSERM, Montpellier, France; 2 Fondation Fondamental, Créteil, France; 3 Department of Mental Health, Fondazione IRCCS, San Gerardo dei Tintori, Monza, Italy; 4 Pôle de Psychiatrie, Centre Hospitalier Princesse Grace, Monaco; 5 Centre Hospitalier Charles Perrens, Laboratoire NutriNeuro (UMR INRA 1286), Université de Bordeaux, Bordeaux, France; 6 Département de Psychiatrie et de Médecine Addictologique, AP-HP, GH Saint-Louis – Lariboisière – Fernand Widal, Pôle Neurosciences Tête et Cou, Université Paris Cité, Paris, France; 7 Department of Psychiatry, CHU Montpellier, Montpellier, France; 8 AP-HP, Groupe Hospitalo-Universitaire AP-HP Nord, DMU ESPRIT, Service de Psychiatrie et Addictologie, Hôpital Louis Mourier, Colombes, France; 9 UMR1266, Université de Paris, Inserm, Sorbonne Paris Cité, Faculté de Médecine, Paris, France; 10 Unité de Recherche Clinique, EPS Ville-Evrard, Neuilly-sur-Marne, France; 11 Service de Psychiatrie de l’Adulte, CIC-1431 INSERM, CHU de Besançon, Laboratoire de Neurosciences, UFC, UBFC, Besançon, France; 12 Pôle de Psychiatrie, Assistance Publique Hôpitaux de Marseille, Marseille, France; 13 Institut de Neurosciences de la Timone, Aix-Marseille Univ, CNRS, Marseille, France; 14 Univ Paris Est Créteil, INSERM, IMRB, Translational Neuro-Psychiatry, AP-HP, DMU IMPACT, FHU ADAPT, Psychiatry and addictology of Mondor University Hospital, Créteil, France; 15 Université Grenoble Alpes, Inserm, U1216, CHU Grenoble Alpes, Grenoble Institut Neurosciences, Grenoble, France; 16 Centre Hospitalier de Versailles, Service Universitaire de Psychiatrie d’Adulte et d’Addictologie, Le Chesnay, France; 17 Bipolar Disorder Expert Centre, Le Vinatier Hospital, Bron, France; 18 University Lyon 1, Villeurbanne, France; 19 Lyon Neuroscience Research Center, Lyon, France; 20 Centre Hospitalier de Versailles, Service Hospitalo-Universitaire de Psychiatrie d’Adultes et d’Addictologie, Le Chesnay, France; 21 Faculté de Médecine Paris-Saclay, Université de Versailles Saint-Quentin-En-Yvelines, Université Paris-Saclay, Villejuif, France; 22 CHU Clermont-Ferrand, CNRS, University of Clermont Auvergne, Clermont-Ferrand, IP, France; 23 Centre Psychothérapique de Nancy, Université de Lorraine, Nancy, France; 24 Service Hospitalo-Universitaire de Psychiatrie Générale et de Réhabilitation Psycho Sociale 29G01 et 29G02, CHRU de Brest, Hôpital de Bohars, Brest, France; 25 Service de Psychiatrie et de Psychologie Médicale, Centre Expert Dépression Résistante FondaMental, CHU de Toulouse, Hôpital Purpan, ToNIC Toulouse NeuroImaging Centre, Toulouse, France; 26 Département de Psychiatrie, Université de Toulouse, Toulouse, France

**Keywords:** biomarkers, bipolar disorder, cognition, cohort, staging, suicide

## Abstract

**Introduction:**

Suicidal behaviors (SB) in bipolar disorder (BD) are major adverse outcomes that may influence disease progression. While staging models exist for psychiatric disorders, none include suicide. This study aims to stratify suicidal risk in BD, propose a staging model for SB, and assess its clinical utility.

**Methods:**

Participants from the FondaMental Advanced Centers of Expertise for Bipolar Disorder (FACE-BD) cohort were categorized into five stages (St) based on SB: St0 (no suicidal ideation [SI]), St1 (SI but no suicide attempt [SA]), St2a (non-severe/violent SA), St2b (severe /violent SA), and St3 (multiple SAs). Stages were analyzed based on demographic, clinical, cognitive, and biological characteristics using logistic regression.

**Results:**

Key differences emerged between stages. St1 showed longer untreated illness and higher lability and lower functioning than St0. St2a was linked to anxiety, substance use disorders, and longer disorder duration, while male gender and lithium bitherapy were protective. St2b exhibited shorter untreated illness and higher childhood trauma (CTQ) scores, with male gender and alcohol use as risk factors. St3 was associated with BD-II, alcohol use, longer disorder duration, and more depressive episodes, but less anxiety. No biochemical or cognitive differences were found across stages. The model was significantly associated with SA occurrence (LRT = 28.74, *p* < 0.0001).

**Conclusions:**

This staging model for suicidality in BD provides a multifaceted approach to risk stratification and predictive insights, although further refinement is needed.

## Introduction

Suicide is a major clinical and public health concern, claiming more than 700,000 lives annually and ranking among the top ten causes of death globally, particularly affecting the 15–34 age group [1–3]. Within this broader context, bipolar disorder (BD) contributes to 3–14% of all suicide-related deaths, with approximately one-third to one-half of patients with BD attempting suicide at least once in life [3, 4].

Identifying BD-specific suicide risk factors is challenging but crucial for risk mitigation, therapy selection, prognosis, clinical research, and drug development [2, 3]. These risk factors encompass both static (non-modifiable) factors, like genetic vulnerability or trauma history, and dynamic (modifiable) factors, representing internal or external circumstances that can change over a lifetime [5]. For mood disorders, particularly BD, a prior suicide attempt (SA) is the strongest predictor of future suicidal behavior (SB), with a 1.6% risk of suicide within the first-year post-attempt, rising to 16% for subsequent attempts [2].

The concept of staging, initially developed for medical chronic conditions, classifies the disease into identifiable stages, guiding prognosis, prevention, and treatment [6–8]. Moreover, a standardized and reliable staging approach can be employed in clinical settings worldwide [9]. In psychiatry, staging is a recent introduction, facing challenges due to the limited understanding of psychiatric disorders’ etiopathogenesis, heterogeneity, and evolutive symptoms, as well as lack of reproducible biomarkers [10, 11]. Moreover, linear progression may not apply universally [12]. To date, without reaching a validated model, clinical staging models have been proposed for psychiatric disorders, such as schizophrenia, Major Depressive Disorder (MDD), and BD [12, 13]. Several potential variables have been identified, but these models do not include suicide in staging nor use suicide as the main classifier for staging [12]. Suicide is not directly correlated with severity of the disorder, and increasing evidence suggests that SB may be a specific entity [14]. There is an urgent need to develop a focused understanding of SB and to improve its prediction in the context of psychiatric conditions that are highly associated with suicide risk, such as BD. Moreover, the presence of SB may influence the course of BD [3]. Regarding suicide prediction, there are different modeling efforts: first-generation models based on clinical judgment and expert opinion, second-generation models incorporating static risk factors, and third-generation models adding dynamic risk factors [5]. However, challenges arise in defining elevated-risk conditions, in identifying dynamic factors associated with acute risk, and in understanding the impact of protective factors [5]. In fact, these factors may vary between individuals and stages [3, 7]. In addition, research on biomarkers and suicide correlation is limited, with a few showing specificity [15, 16]. Literature explores factors related to suicidal ideation [17–20], single SA [21–24], and comparisons of patients with violent or multiple SA [25–28], but these conditions are rarely studied together. Clinical staging models in suicidal individuals with BD are underexplored and proposed staging models are mostly theoretical and need validation through clinical studies [9, 12].

In light of these unmet needs, the aim of our study is among patients suffering from BD (1) to stratify the suicidal risk, (2) to identify a possible staging model of SBs and (3) to investigate the potential clinical use of this model. Indeed, we hypothesize that a staging model based on both static and dynamic factors might provide clinicians with a useful tool to predict the course of both SB and BD, particularly organizing treatment strategies to prevent SBs.

## Material and methods

### Participants enrolment

This observational multicentric study was conducted in the cohort FACE-BD (FondaMental Advanced Centers of Expertise for Bipolar Disorder). This national network, coordinated by Fondation FondaMental, is constituted by 14 centers located in different regions of France, where patients affected by BD are referred by their general practitioner or a psychiatrist or by patient associations to be evaluated and receive care [29]. The main aim is to provide comprehensive and standardized assessments as well as an evidence-based and personalized treatment approach to people with BD, while the research component was subsequently added to this clinical framework [30]. All outpatients that meet DSM-IV criteria for BD, subtype I, II or Not Otherwise Specified, can be included in the cohort [31]. On the other side, exclusion criteria consist of ongoing severe mood episode, which would make evaluation impossible, dementia-related disorders, or intellectual disability [29]. At baseline, patients undergo an evaluation conducted by an expert multidisciplinary team composed of nurses, psychologists, neuropsychologists, and psychiatrists, who also confirm the diagnosis of BD using the Structured Clinical Interview for DSM-IV Axis I Disorders [32]. Sociodemographic and disorder characteristics, SB history, psychiatric comorbidities, clinical dimensions, treatment history, as well as psychiatric family history and history of somatic comorbidities are collected [30]. The assessment also includes physical examination with routine blood tests and a cognitive battery administration [29]. Patients are followed semi-annually or annually [29]. In accordance with French regulations, patients were informed and provided written non-opposition consent, which is the standard procedure for research conducted within the context of routine care. The assessment protocol was approved by the ethical committee (Comité de Protection des Personnes – Île de France IX, 18 January 2010). The complete methodology has already been described elsewhere [29–31].

### Patients assessment

From the FACE-BD cohort, we extracted data from 4754 participants, recruited between 1 January 2009 and 31 May 2022. During the clinical interview, sociodemographic data (age at inclusion, sex, education level) and clinical characteristics were assessed. These included: bipolar subtype, age at onset, number of depressive, (hypo)manic, and mixed episodes (defined according to DSM-IV criteria), polarity at onset (elevated, namely hypomanic/manic/mixed, or depressive), presence of a rapid cycling pattern, lifetime psychiatric comorbidities (anxiety disorders, substance use disorder [SUD: cannabis, cocaine], and alcohol use disorder [AUD]), age at first psychotropic treatment, smoking status, number of psychiatric hospitalisations, treatment, illness duration, and duration of untreated illness (DUI), defined as the time between illness onset and the administration of a proper treatment, that is guideline-recommended [33]. History of suicide ideation (SI) was evaluated with the following questions: “Have you ever wished to be dead?,” to assess passive ideation, “Have you ever thought to kill yourself even if you will never do it?” and “Have you ever planned to take your life?,” for the active ideation. Lifetime history and number of SA were recorded, while interrupted and aborted attempts as well as non-suicidal self-aggressive behavior were not included. Moreover, lifetime history of severe (conditioning admission to intensive care) and violent SA, defined according to Asberg’s criteria (hanging, jumping from heights or under a train, car crash, use of firearms, burning, gas poisoning, electrocution, drowning, and several deep cuts) were reported by the clinician [34].

Moreover, scales were administered to evaluate current manic (Young Mania Rating Scale [YRMS]) [35] and depressive symptoms (Montgomery-Asberg Depression Rating Scale [MADRS] and Quick Inventory of Depressive Symptoms-Self-Rating [QIDS-SR]) [36, 37]. Current anxiety was assessed using Spielberger State – Trait Anxiety Inventory (STAI version Y-A) [38], while emotional reactivity was assessed using Affective Lability Scale (ALS) [39]. Childhood history of Adult Attention Deficit Hyperactivity Disorder (ADHD) was evaluated through the Wender Utah Rating Scale (WURS) [40] and childhood history of trauma through Childhood Trauma Questionnaire (CTQ) [41]. Sleep disturbances were assessed using the Pittsburgh Sleep Quality Index (PSQI) [42], while functioning using the Functioning Assessment Short Test (FAST) [43]. Finally, impulsivity was measured using the Barrat Impulsiveness Scale version 10 (BIS-10) [44] and adherence to treatment using the Medication Adherence Rating Scale (MARS) [45]. Elevated total scores indicate greater severity in all cases, with the exception of the latter, in which a high rating means a good compliance to treatment. Current psychotropic treatment was recorded.

### Biological factors

A medical examination was performed by a healthcare professional to measure the body mass index (BMI), with body weight and height, and waist circumference. Moreover, a blood sample was taken in the morning, after overnight fasting, to measure: complete blood count, total cholesterol, high-density lipoprotein (HDL), low-density lipoprotein (LDL), triglycerides, total bilirubin, albumin, urate, and thyroid-stimulating hormone (TSH). In addition to white blood cells, other indicators of inflammatory status were measured: C-reactive protein (CRP), NLR (neutrophil-to-lymphocyte ratio), PLR (platelet-to-lymphocyte ratio), and MLR (monocyte-to-lymphocyte ratio).

### Cognitive battery

Standardized cognitive tests were performed to evaluate the following cognitive domains: (1) verbal episodic memory, using the California verbal learning test (CVLT) free immediate recall [46]; (2) working memory, using the Digit Span subtest from the Wechsler Adult Intelligence Scale version III (WAIS-III) [47]; (3) processing speed, using the Digit Symbol Coding subtests of WAIS-III and the Trail Making Test (TMT) part A [48]; (4) executive functions, using the color/word condition of the Stroop test, the TMT part B and Verbal Fluency (semantic and phonological) [49].

### Statistical analysis

Five stages were defined *a priori*, based on the exploration of literature:

- St0: no lifetime history of active SI,
- St1: history of SI but no history of SA,
- St2: history of one SA,
  - St2a: neither violent nor severe SA,
  - St2b: violent or severe SA,
- St3: history of more than 1 SA (whatever severity or violence of SA).

The sample distribution of subjects across stages is *N*_St0_ = 1427, *N*_St1_ = 1664, *N*_St2a_ = 522, *N*_St2b_ = 238, *N*_St3_ = 903.

Stages were characterized for each sociodemographic and clinical component, as well as for treatment, by their means with standard deviation (SD) or their percentage distribution across levels according to the quantitative or categorical nature of the variable. Global significance of the differences found between the stages for each feature was tested using respectively *F*-test (univariate ANOVA) or Chi Square test for quantitative and qualitative variables. When they did not match the normality assumption, quantitative variables were log transformed or turned to qualitative using tercile cutoffs, allowing balanced sample size between levels. *p*-Values were Bonferroni corrected for multiple testing. Subsets of variables with association reaching a significance of alpha <0.2 are recorded and selected for further analysis.

A multivariate analysis was performed using a multinomial regression model to assess independent characteristics of stages from the set of variables selected in the univariate analysis. Variable Step Selection procedure (backward) with *p*-value threshold set to 0.2 was applied to keep only meaningful variables and to prevent collinearity. Logistic regressions were then used to test differences between stages according to following five contrasts: St0 vs St1, St1 vs St2a, St1 vs St2b, St2b vs St2a, and St2a or St2b vs St3. Likelihood Ratio Test (LRT), OddsR, and 95% confidence intervals were computed. As quantitative variables were *z*-scored, OddsR may be interpreted as an effect size. Missing data were handled by using a listwise deletion approach.

Due to their specificity and to keep a maximum of participants, biological, cognitive, and inflammatory (blood cells count and CRP) sets of variables were treated separately in additional analysis. We use MANOVAs to test if stages show global differences on z-scored variables. Models were adjusted for sex, age, education, lifetime anxious disorder, substance use, alcohol use, and treatment. For the biology and inflammatory sets, the model was also adjusted for smoking status and BMI. For the inflammatory set, subjects with CRP > 15 mg/L were removed from the analysis. Post-hoc ANOVAs were performed for each component separately when MANOVA reported a global signal and marginal means, and 95% confidence intervals were computed.

We tested the predictive power of the staging model by testing if stages were associated with future SA during a 12-month follow-up. We compared a model with adjusting variables (sex, age, education, bipolar type, lifetime anxious disorder, lifetime SUD, and AUD) with the same model but involving stages variable. We also computed ROC curves, AUC, accuracy, sensitivity, and specificity.

For all tests, we considered a 5% limit threshold (*p*-value <0.05). Analyses were performed using R software version 4.1.2 (2021-11-01).

## Results

Among the 4754 patients included in the univariate analysis, the majority were women (62.0%), with a mean age of 39.7 (13.1) years, equally divided between BD type I and type II.

### Sociodemographic and clinical variables

**Figure 1.**
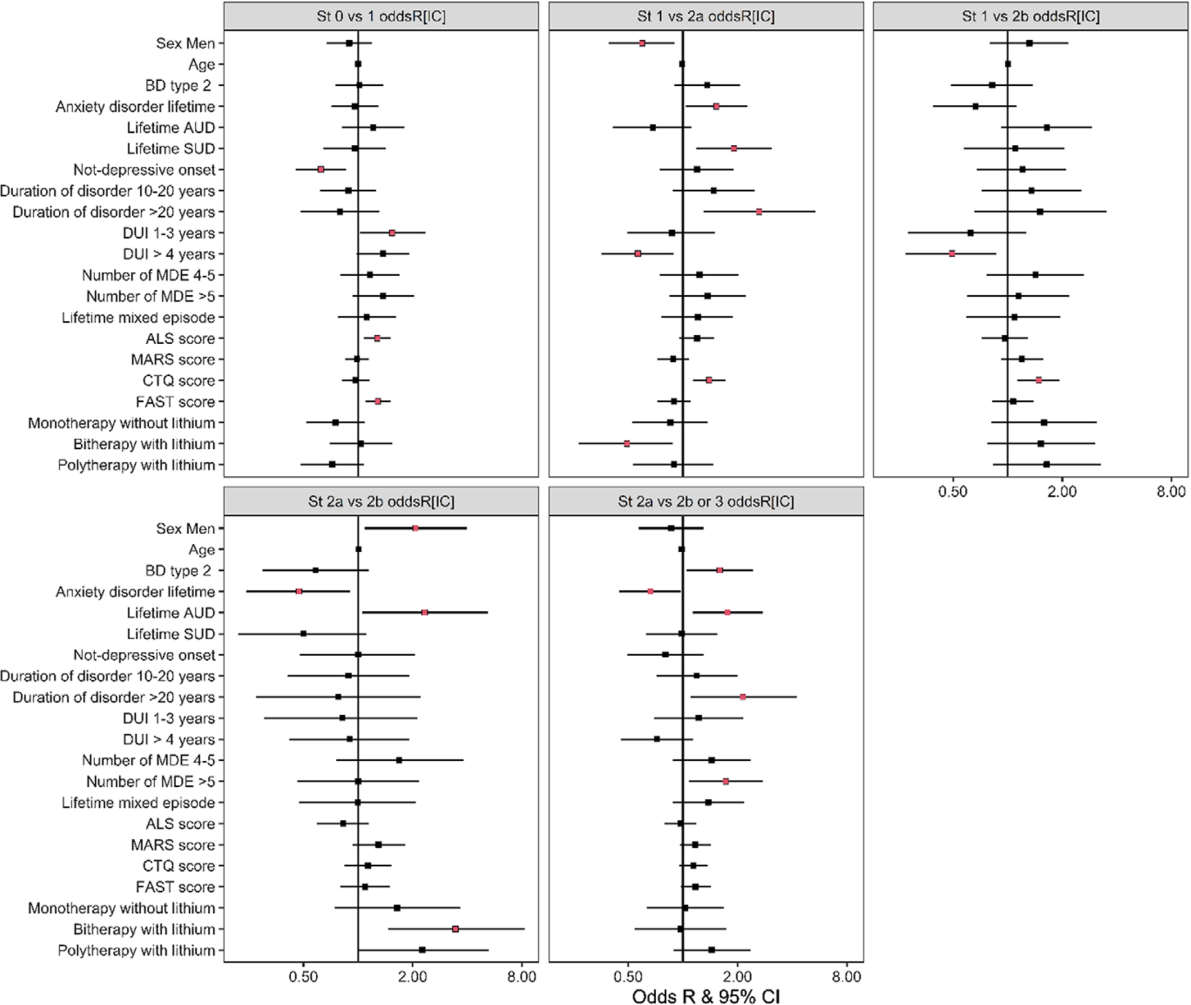
Tree plot presenting OddsR and 95% confidence intervals, estimated from the multinomial regression analysis after the StepAIC selection procedure for each meaningful contrast. Red square along with confidence intervals illustrating the significance of the effects. Reference levels are women, bipolar type 1, no lifetime anxious disorder, no AUD or SUD, depressive first episode, lower terciles for duration of the disorder (with or without treatment) and number of MDE, no lifetime occurrence of mixed episode, and polytherapy without lithium.

From the univariate exploration (Table 1 in Supplementary material) only sex, age, education level, bipolar types, current anxious disorder, alcohol and substance use disorder, rapid cycling, polarity at onset, duration of the disorder, DUI, number of depressive, maniac, hypomanic or mixed episode, smoking status, affective lability (ALS), medication adherence (MARS), childhood maltreatment (total CTQ score), depression (MADRS), sleep quality (PSQI), functioning (FAST) and class of treatment were included in the multinomial regression. After listwise deletion, 1443 individuals are still involved in the analysis (*N*_St0_ = 372, *N*_St1_ = 561, *N*_St2a_ = 168, *N*_St2b_ = 87, *N*_St3_ = 255). Meaningful independent associations characterizing differences between stages which remain after passing through the Backward selection procedure are presented in Figure 1.

When contrasting St0 and St1 (SI), St1 showed a lower proportion of (hypo)manic and mixed index episode (OddsR = 0.624[0.453;0.858]) than depressive index episode, longer DUI (OddsR_1–3_ = 1.537[1.017;2.348], OddsR_4–51_ = 1.373[0.984;1.919]), higher scores for ALS (OddsR = 1.276[1.08;1.512]), and FAST (OddsR = 1.283[1.093;1.511]). Regarding differences between St1 and St2a (non violent non severe SA), the latter exhibited greater presence of a comorbid anxiety disorder (OddsR = 1.528[1.032;2.264]) and SUD (OddsR = 1.906[1.179;3.077]), longer duration of the disorder (OddsR_10–20_ = 1.473[0.882;2.474], OR_21–68_ = 2.629[1.306;5.387]) as well as a shorter DUI (OddsR_1–3_ = 0.87[0.494;1.498], OddsR_4–51_ = 0.565[0.357;0.885]). Male gender (OddsR = 0.596[0.389;0.902]) and bitherapy with Lithium (OddsR = 0.492[0.267;0.875]) were protective for SA when contrasting with polytherapy without lithium, while a higher score for CTQ was a risk factor for SA (OddsR = 1.393[1.137;1.71]).

St1 and St2b (one violent or severe SA) were differentiated by DUI, which seemed shorter for St2b (OddsR_1–3_ = 0.623[0.281;1.268], OddsR_4–51_ = 0.493[0.273;0.868]), and CTQ score (higher in stage 2b, OddsR = 1.483[1.135;1.934]). When contrasting St2a and St2b, male gender (OddsR = 2.071[1.085;3.961]) and AUD (OddsR = 2.321[1.058;5.207]) were risk factors for violent/severe SA, while St2a showed a higher proportion of patients with anxious disorder (OddsR = 0.472[0.243;0.9]) and fewer receiving a bitherapy with Lithium (OddsR_lith, 1 other_ = 3.438[1.463;8.29], OddsR_lith, 1 or 2 others_ = 2.254[0.987;5.25]).

St2a and St2b differed from St3, since BD-II (OddsR = 1.598[1.052;2.44]) and AUD (OddsR = 1.761[1.131;2.76]) appeared to be risk factors for repeated SA, as well as a longer duration of the disorder (OddsR_10–20_ = 1.19[0.714;1.993], OddsR_21–68_ = 2.137[1.098;4.226]) and higher number of depressive episodes (OddsR_6–20_ = 2.137[1.098;4.226]), while anxiety disorder seemed less represented in St3 (OddsR = 0.662[0.447;0.975]).

### Biological parameters and cognition

**Figure 2.**
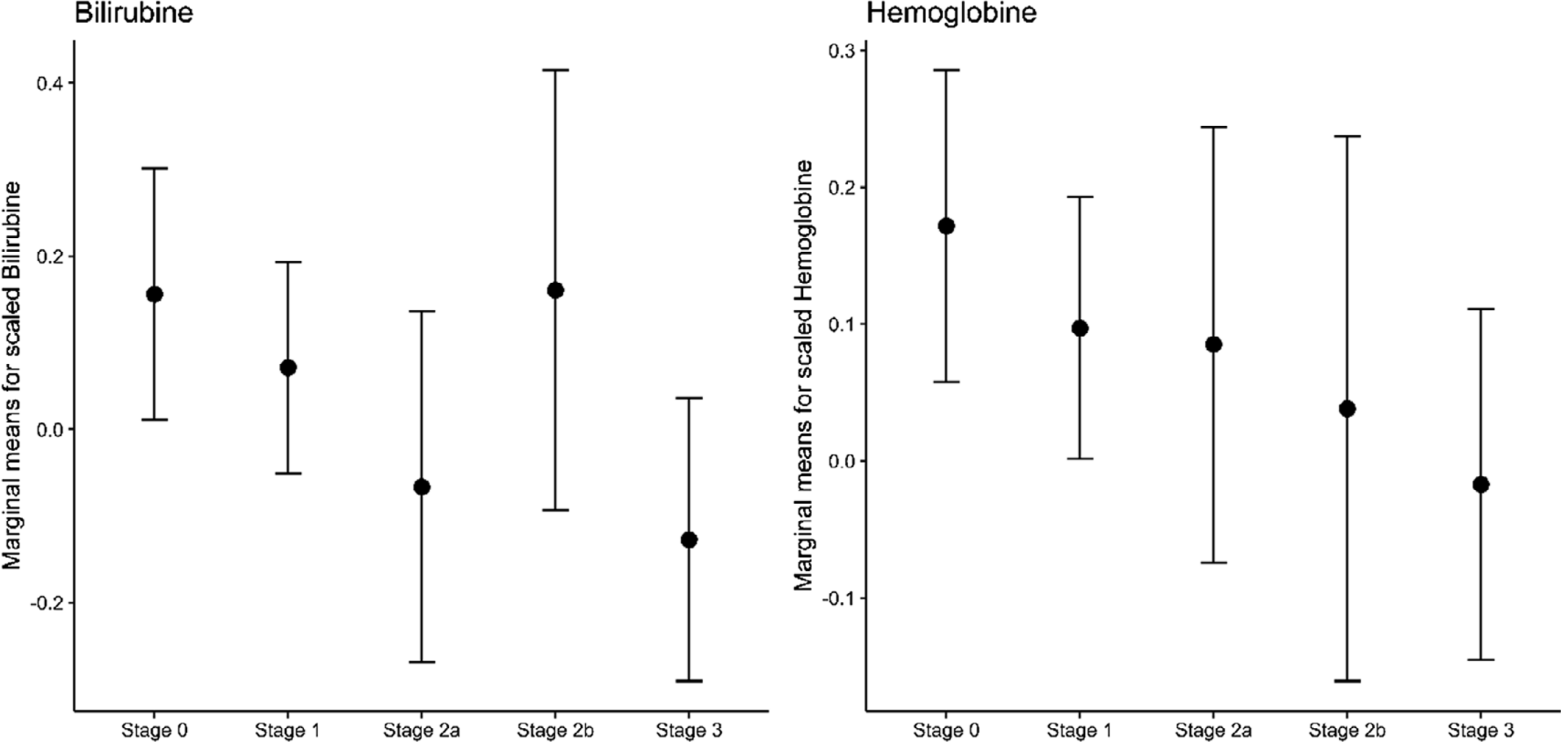
Adjusted marginal means for biological variables across stages and their 95% confidence intervals. Only significantly associated variables are presented.

There was no significant between-stages difference in terms of white cell count, platelets, CRP, NLR, PLR, and MLR (Table 1). Marginal between-stages difference was found for biological components (*p* = 0.05). While there was no significant difference between stages concerning albumin, cholesterol (total, HDL and LDL), triglycerides, TSH, and urate, hemoglobin appeared to be lower in St3 than in St0 and St1 (*p* = 0.03), and Bilirubin levels were lower in St2a and St3 than in St0 and St1 (*p* = 0.001), as shown in Figure 2 (Table 2 in Supplementary material). No significant cognitive differences were observed between stages. Both univariate analyses on individual cognitive tests and multivariate approach treating cognition as a global construct did not reveal significant associations with the stages (Table 1 in Supplementary material and Table 1).

**Table 1.** Results of MANOVAs testing for between stages differences for each set of variables (cognition, biology, and inflammation) separately

	Df	Pillai	Approx *F*	Num Df	den Df	Pr(>*F*)
Cognition,^a^ *N* = 600	4	0.0611	0.747	48	2312	0.9
Biology,^b^ *N* = 1615	4	0.0344	1.384	40	6380	*0.05*
CRP and WBC,^b^ *N* = 2404	4	0.0045	1.36	8	4780	0.21
Cell counts,^b^ *N* = 2404	4	0.006	0.602	24	9552	0.94
Cell count ratios,^b^ *N* = 2404	4	0.0026	0.51	12	7170	0.91

a *Model* adjusted for sex, age, education, AUD, SUD, and treatment.

b Model adjusted also for smoking status and BMI.

*p* < 0.05.

### Predictivity

The predictivity of our *a priori* staging model was tested by examining whether the stages correlated with the occurrence of SA within the subsequent 12 months of follow-up. In the total sample of 764 patients having data at one-year follow-up, 34 individuals attempted suicide. Staging was significantly associated with the occurrence of SA (LRT = 28.74, Df = 4, *p* < 0.0001): in particular, St2b and St3 presented an increased risk with OddsR respectively equal to 7.600[1.319; 43.764] and to 11.443[3.548; 51.495]. OddsR were adjusted for sex, age, education, bipolar type, depression, anxious disorder, SUD, and AUD. Including stages in the model noticeably increased the AUC from 0.75 to 0.85. Nevertheless, Accuracy was rather moderate (0.77), mainly due to moderate Specificity (0.77), while Sensitivity amounted to 0.82. As highlighted by Positive Predictive Value, equal to 0.14, this model detected lots of false positives, while the Negative Predictive Value was up to 0.99.

## Discussion

To the best of our knowledge, this is the first study to explore the staging of suicide in BD in relation with a large set of clinical variables and which finds different risk and protective factors associated with stages: gender, duration of the disorder and DUI, type II BD, number of depressive episodes, depressive onset, treatment including lithium and comorbidities such as anxiety disorder, SUD and AUD. Likewise, we noted that the stages differed in ALS, FAST, and CTQ scores, as well as in the level of few biological variables (bilirubin, hemoglobin). Moreover, owing to the multicentric nature of the FACE-BD cohort, we have been able to collect these data from a large sample of outpatients who suffer from BD.

Our results showed that, in agreement with previous publications, female gender seems to be a risk factor for SA (stage 1 vs 2a), while men appear more likely to make a violent SA (stage 2b) [3, 50–52].

We found that longer duration of the disorder was a risk factor for SA and, especially, for multiple SA, in line with previous findings [27]. Unexpectedly, we observed that a longer DUI was associated with a higher risk of SI, while a shorter DUI was associated with SA. Various studies in literature have noted that individuals with untreated BD for more than two years exhibited a significantly higher frequency of SA compared to those with two years or less of untreated illness [53, 54]. One possible explanation could be that those who attempt suicide hopefully access medical care more promptly.

In line with previous results, we found that type II BD as well as a higher number of depressive episodes were risk factors for repeated SA (St3). An explanation could be found in the fact that, as reported by Forte and colleagues (2015), depressive state accounted for three quarters of the percentage of time ill among treated subjects with BD, exposing them to a protracted and sustained suicidal risk [55, 56]. Additionally, the treatment of bipolar depression is less effective, partly because of the use of antidepressants that may induce or worsen mixed episodes [2]. Nevertheless, to date, there is a lack of agreement on this issue: in fact, various studies reported similar rates in the two BD types [57, 58], while others pointed out more elevated rates of SA in type I BD [52]. In particular, certain authors highlighted an association between type I BD and violent SA [59].

Moreover, an elevated-mood onset (hypomanic/manic/mixed) appeared to be associated with a lower likelihood of SI (stage 1). This finding aligns with previous studies suggesting that depressive onset may be linked to a higher rate of SA [60–62]. This result might be partly related to the fact that a manic episode is so striking that it is promptly intercepted, increasing the possibilities of being adequately treated, to avoid more severe consequences. Other authors also hypothesized a mediation by grandiose optimism or less hopelessness in manic patients [62]. However, it should not be forgotten that we do not have the data about the deaths by suicide [58, 63]. In addition, insight is more compromised in mania than in hypomania or depression, although this may lead to opposite implications, as highlighted by conflicting findings in literature: on the one hand, lack of insight may result in a poor adherence to treatment, with a consequent increase in the risk of relapses and of SA [64]. On the other hand, a greater insight might be related to higher SI due to patients’ awareness of their malaise [65, 66]. However, a lack of agreement persists on this issue [67, 68].

With regard to comorbidities, an anxiety disorder seemed to be a risk factor for SA (St2a), but protective for violent SA. Preti and colleagues underlined how patients suffering from BD and comorbid anxiety disorders showed a higher proportion of depressive episodes and were more frequently treated with benzodiazepines [69]. Exposure to such treatment is itself a risk factor to both SA and suicide [70]. Moreover, several studies reported an association between anxiety disorders and an overall severity of BD, possibly in connection with sleep disturbances, enhanced mood instability, and increased risk of substance abuse [71, 72]. However, patients suffering from anxiety disorders seemed less likely to repeat SA (St3), partially in contrast with Arici and colleagues’ findings [27]. A lifetime history of SUD appeared to be a risk factor for SA (St2a), while a lifetime history of AUD seemed to be a risk factor for both violent SA (St2b) and multiple SA (St3). This result points out a possible link with impulsivity, which was found to be related to SA in various studies, possibly for neurocognitive alterations due to substances misuse: in fact, substances misuse not only favors mood instability and impulsivity, but also amplifies neurocognitive alterations with a consequent increased risk of SB [53, 68, 73–75]. In particular, Etain and colleagues (2013) pointed out higher BIS-10 total scores in patients with alcohol or cannabis misuse, but, like ourselves, they did not observe an association with previous SA [76].

In addition, higher scores in affective lability, assessed using ALS, seemed to be a risk factor for SI (St1), as previously shown [77, 78].

Furthermore, history of childhood trauma was a risk factor for SA (both St2a and St2b) in line with other works in literature, which found greater frequency of SA in patients suffering from BD with high total CTQ score [2, 79–81]. Interestingly, Cazala et al. (2019) pointed out a different effect of physical abuse with respect to gender: in fact, women appeared to be more likely to exhibit SB, while men were more likely to develop irritability [82]. Childhood trauma might impair the capability to regulate emotions and impulsivity: consequently, impulsivity and affective lability might be mediators of the association between childhood trauma and suicidality [77, 83, 84].

Additionally, poor functioning levels were a risk factor for SI but not for SA. Although there is a lack of both agreement and studies in literature about the impact of functioning on SB, some authors found an association between limited global functioning and suicidality, as well as severe mixed and depressive symptoms and childhood trauma [85–89]. Moreover, Almeida and colleagues suggested that attempters might have functional alterations due not to a direct effect, but because of some clinical mediators, such as greater impulsiveness or current depressive symptoms [90].

With respect to treatment, we observed that bitherapy with lithium was protective for SA when compared with polytherapy without lithium, in line with findings in literature [4]. In fact, lithium seems to carry the greatest clinical efficacy not only for mood stabilization and anti-impulsive effects but also for its distinct and independent anti-suicidal properties, resulting in an estimated risk reduction of over 60% [4]. This effect holds true even for moderate and poor responders, although the precise mechanism remains unclear to date [15, 91]. When compared with St2a, St2b showed a higher proportion of patients receiving a bitherapy with lithium: this trend likely stems from the introduction of lithium treatment in these patients given their medical history of previous violent or severe SA.

As regards biological parameters, we noted a reduction in bilirubin among patients in advanced stages. Since it is an endogenous antioxidant, it might indicate an alteration of the defenses against oxidative stress, not only in bipolar patients, but in particular in those with a positive history of one or more SA (St2–3), as other studies have also suggested [53]. We found no differences between stages concerning uric acid, though some studies have noted an inverse correlation between uric acid levels and the severity of SI in individuals with major depression or BD, potentially owing to its connection with purinergic transmission and antioxidant activity [92, 93]. We also found no association with the lipid profile, about which literature has produced mixed findings [26, 50, 94, 95].

With regard to hemoglobin, we found diminished levels among patients in advanced stages. This seems in line with other studies: for example, Memic-Serdarevic and coauthors found low hemoglobin concentration in BD as compared to healthy controls, both at baseline and after treatment [96]. In fact, since it is indicative of blood oxygenation, hemoglobin has been associated with an increased risk of different psychiatric conditions such as affective disorders [96–100]. According to some investigators, a lowered hemoglobin in BD might also be associated with an inflammatory state, since many systemic cytokines inhibit erythropoiesis [99]. However, on the other hand, very few studies have explored the relationship between hemoglobin levels and SA, but no evidence of an association has been found [101].

In this light, with regard to inflammation, we explored a possible association with different markers, namely NLR and PLR, which may increase in relation to both BD and to SB in patients with unipolar or bipolar depression [102, 103]. A meta-analysis suggests that this increase may depend on the phase of BD, as no significant differences were observed between euthymic bipolar patients and healthy controls [103]. Therefore, it might be inferred that, while inflammation is significant across all phases of BD, the inflammatory response tends to be less pronounced during euthymic phases. This could explain why we found no statistically significant differences in our sample, which consisted mainly of outpatients, euthymic or with mild-to-moderate symptoms. Consistent with this, we observed no significant difference in CRP levels among the stages: in fact, according to some studies, CRP might be related to suicide risk, and, in particular, to recent suicide behavior, hypothesizing it to be a state marker [104, 105]. Nevertheless, biological parameters should be measured non-retrospectively in order to identify markers characteristic of each stage.

As regards cognitive variables, we failed to find an association with stages, despite previous studies showing an association between SA, and in particular violent SA, and poorer verbal memory performance [106, 107]. Furthermore, other researchers highlighted an impairment of working memory and inhibitory control in patients suffering from BD or with a history of SB [108, 109].

Finally, in line with our hypothesis, the results support the predictive validity of our model in identifying individuals at risk of SA within one year. The improvement in AUC after including the staging variable supports its added value in risk prediction, although the model needs further refinement to improve its accuracy and reduce unnecessary clinical alerts due to the high rate of false positives.

In conclusion, applying a staging approach to suicidality in BD enables the identification of different phenotypes, each with distinct risk factors for progression to more severe stages. Moreover, it offers a structured and multidimensional framework for improving the care of individuals with BD who are at risk of SB [110]. In fact, it encourages clinicians to explore a wider range of factors, markers and stage specifiers than the standard DSM criteria, to identify precociously individuals at different risk levels and, therefore, to intervene proactively, providing appropriate support to prevent SB from escalating and tailoring treatment plans to the specific needs of individuals at different risk stages [9].

This approach allows for targeting at-risk stages with preventive interventions, such as psychoeducation, substance abuse prevention, addressing affective lability or anxiety disorders, and assessing childhood trauma [110]. In contrast, patients at more advanced stages may require more intensive interventions, including psychopharmacotherapy with anti-suicidal efficacy and safety planning [110].

A risk stratification also allows more effective allocation of resources given their scarcity in the mental health field [111]. The application of a staging approach to suicidality might enhance clearer communication among healthcare personnel, patients, and caregivers, facilitating shared decision-making. This also extends to research, helping a more comprehensive understanding of the factors associated with the evolution of SB in this population. At the same time, it might offer the possibility of assessing treatment efficacy and identifying novel interventions to reduce suicide risk, in a more standardized framework.

### Limitations

This study has several potential limitations: first of all, we could construct the staging model through an *a priori* approach, based solely on lifetime data, preventing patients from regressing to lower stages. We tested the possibility of performing the same analysis, taking into account the preceding year, but the number of SA was too small for meaningful analysis. In this sense, the model might overestimate relapses because of the lack of information about the variations of clinical variables in the short term, which would be interesting to include in the analysis to improve the predictivity of SA. Additionally, interrupted and aborted attempts as well as the frequency and intensity of SI were not assessed, as the Columbia Suicide Severity Rating Scale was introduced only recently in the cohort assessments. It is also possible that, in our cohort, patients with highly severe BD are underrepresented, for example since they could not be evaluated because of a chronic hospitalization, as well as subjects stabilized with a more favorable clinical progression, who might be not referred to FACE-BD network [87]. Furthermore, SA might be underestimated in our sample, as these individuals are in care and monitored, not only by our network but hopefully also by caregivers. These factors could possibly interfere with the generalisability of our results. In addition, as mentioned above, since the cohort consists of routine-care patients, there are lots of dropouts during follow-up, further impacting longitudinal analyses. Importantly, the initial diagnostic assessments in the cohort were based on DSM-IV criteria. As a result, the classification of mixed episodes may differ from current DSM-5-TR definitions, potentially affecting the interpretation of onset polarity and its association with suicidality. Finally, as previously mentioned, the moderate specificity and low positive predictive value of our model led to a considerable number of false positives, which may limit its clinical applicability in real-world settings.

## Conclusions

In this study, we proposed a staging model for suicidality in BD, with stages showing significant associations with a broad range of sociodemographic, clinical, and some biological features. While the approach is innovative, it requires refinement to address the limitations of a lifetime-based model. Future research will focus on exploring alternative methodologies, adopting a longitudinal perspective, and incorporating genetic factors to enhance the model’s robustness. Additionally, replication in independent samples and validation using external and objective measures will be essential to confirm its utility and reliability. Finally, although data on suicide fatalities are not available in the current cohort, incorporating such information in future studies could provide a more comprehensive assessment of extreme risk profiles and further strengthen the clinical relevance of the staging model.

## Supporting information

10.1192/j.eurpsy.2025.10068.sm001 Auxilia et al. supplementary material 1 Auxilia et al. supplementary material

10.1192/j.eurpsy.2025.10068.sm002 Auxilia et al. supplementary material 2 Auxilia et al. supplementary material

## Data Availability

The data that support the findings of this study are available under request.

## Supplementary material

The supplementary material for this article can be found at http://doi.org/10.1192/j.eurpsy.2025.10068.
