# Supplementary material for: Staging of suicidality in bipolar disorder: Findings from the FACE-BD cohort (FondaMental Advanced Centers of Expertise for Bipolar Disorders)
Source: Eur Psychiatry. 2025 Jul 22;68(1):e117. doi: 10.1192/j.eurpsy.2025.10068 (PMC12438995; doi:10.1192/j.eurpsy.2025.10068)
Supplement: Auxilia et al. supplementary material 1 — Auxilia et al. supplementary material [file S0924933825100680sup001.docx]

**Table 1.** Univariate characterisation of the 5 stages evaluated from the whole sample.

| Variables | Levels | Stage 0 mean(sd)/N(%) | Stage 1 mean(sd)/N(%) | Stage 2a mean(sd)/N(%) | Stage 2b mean(sd)/N(%) | Stage 3 mean(sd)/N(%) | p-value | Statistics | Corrected p-value |
| --- | --- | --- | --- | --- | --- | --- | --- | --- | --- |
| ***Socio-demographic features*** |  |  |  |  |  |  |  |  |  |
| Sex | women | 794(55.7) | 947(57) | 378(72.6) | 129(54.4) | 688(76.3) | <0.0001 | chi2 = 150.058 ; df = 4 | <0.0001 |
|  | men | 632(44.3) | 715(43) | 143(27.4) | 108(45.6) | 214(23.7) |  |  |  |
| Age |  | 40.364(13.553) | 39.009(12.961) | 38.897(12.934) | 41.496(12.835) | 39.934(12.649) | 0.005 | F = 3.768 ; df = 4 ; dfres = 4749 | 0.8 |
| Education level (years) | 0-13 | 337(32.5) | 439(31) | 153(36.5) | 71(35.5) | 316(43) | <0.0001 | chi2 = 38.129 ; df = 8 | 0.001 |
|  | 14-16 | 556(53.7) | 768(54.2) | 222(53) | 102(51) | 323(43.9) |  |  |  |
|  | >16 | 143(13.8) | 211(14.9) | 44(10.5) | 27(13.5) | 96(13.1) |  |  |  |
| Marital status | single | 522(49.6) | 733(51.6) | 212(50.5) | 102(50.7) | 410(54.6) | 0.32 | chi2 = 4.673 ; df = 4 | 1 |
|  | couple | 531(50.4) | 687(48.4) | 208(49.5) | 99(49.3) | 341(45.4) |  |  |  |
| ***Clinical characteristics*** |  |  |  |  |  |  |  |  |  |
| Bipolar type | BD type 1 | 724(50.7) | 720(43.3) | 206(39.5) | 119(50) | 343(38) | <0.0001 | chi2 = 47.291 ; df = 4 | <0.0001 |
|  | BD type 2 | 703(49.3) | 944(56.7) | 316(60.5) | 119(50) | 560(62) |  |  |  |
| Mood episode (last year) | no | 284(26.1) | 309(20.7) | 97(21) | 50(22.7) | 132(16.9) | <0.0001 | chi2 = 24.239 ; df = 4 | 0.01 |
|  | yes | 806(73.9) | 1185(79.3) | 366(79) | 170(77.3) | 651(83.1) |  |  |  |
| Anxiety disorder (lifetime) | no | 694(64.1) | 830(55.3) | 195(42.7) | 137(62) | 372(46.2) | <0.0001 | chi2 = 93.543 ; df = 4 | <0.0001 |
|  | yes | 389(35.9) | 671(44.7) | 262(57.3) | 84(38) | 433(53.8) |  |  |  |
| Polarity at onset | MDD | 777(65.2) | 1219(76.5) | 397(79.7) | 168(72.1) | 697(82.4) | <0.0001 | chi2 = 93.808 ; df = 4 | <0.0001 |
|  | Hypo/Mania/mixed | 415(34.8) | 374(23.5) | 101(20.3) | 65(27.9) | 149(17.6) |  |  |  |
| Age at first episode |  | 24.925(10.159) | 24.049(9.381) | 21.981(8.498) | 24.524(9.91) | 21.413(8.497) | <0.0001 | F = 22.277 ; df = 4 ; dfres = 4309 | <0.0001 |
| Duration of the disorder (tercile) | 0-9 | 427(36.2) | 618(39) | 145(29.9) | 68(29.2) | 206(24.8) | <0.0001 | chi2 = 76.781 ; df = 8 | <0.0001 |
|  | 10-20 | 425(36) | 538(33.9) | 167(34.4) | 81(34.8) | 294(35.4) |  |  |  |
|  | 21-68 | 328(27.8) | 429(27.1) | 173(35.7) | 84(36.1) | 331(39.8) |  |  |  |
| DUI (tercile) | 0 | 584(57.1) | 708(50.4) | 220(50.3) | 113(54.3) | 369(51.1) | 0.06 | chi2 = 14.805 ; df = 8 | 1 |
|  | 1-3 | 138(13.5) | 210(14.9) | 57(13) | 29(13.9) | 107(14.8) |  |  |  |
|  | 4-51 | 301(29.4) | 488(34.7) | 160(36.6) | 66(31.7) | 246(34.1) |  |  |  |
| Polarity of last episode | MDD | 574(54.9) | 954(64.1) | 290(64.7) | 142(65.1) | 518(68.7) | 0.0005 | chi2 = NA ; df = NA | 0.09 |
|  | Hypomania | 209(20) | 283(19) | 89(19.9) | 35(16.1) | 112(14.9) |  |  |  |
|  | Mania | 202(19.3) | 161(10.8) | 40(8.9) | 20(9.2) | 43(5.7) |  |  |  |
|  | Mixed | 49(4.7) | 75(5) | 24(5.4) | 19(8.7) | 72(9.5) |  |  |  |
|  | nonspecific | 12(1.1) | 15(1) | 5(1.1) | 2(0.9) | 9(1.2) |  |  |  |
| Rapid cycling | no | 928(88.1) | 1216(84) | 339(76.7) | 174(85.3) | 568(74.6) | <0.0001 | chi2 = 69.597 ; df = 4 | <0.0001 |
|  | yes | 125(11.9) | 231(16) | 103(23.3) | 30(14.7) | 193(25.4) |  |  |  |
| Number of MDD (lifetime, tercile) | 0-3 | 571(56.6) | 647(46) | 168(39.9) | 92(43.4) | 183(27.6) | <0.0001 | chi2 = 156.219 ; df = 8 | <0.0001 |
|  | 4-5 | 216(21.4) | 345(24.5) | 113(26.8) | 57(26.9) | 184(27.7) |  |  |  |
|  | 6-20 | 221(21.9) | 415(29.5) | 140(33.3) | 63(29.7) | 297(44.7) |  |  |  |
| Number of hypomanic episodes (lifetime, tercile) | 0-1 | 453(49.9) | 532(42.9) | 167(44.1) | 71(42) | 219(37.6) | 0.0003 | chi2 = 29.456 ; df = 8 | 0.046 |
|  | 2-3 | 257(28.3) | 371(29.9) | 99(26.1) | 53(31.4) | 186(32) |  |  |  |
|  | 4-20 | 197(21.7) | 337(27.2) | 113(29.8) | 45(26.6) | 177(30.4) |  |  |  |
| Number of manic episodes (lifetime, tercile) | 0 | 552(49.8) | 896(58.4) | 291(62.6) | 112(51.9) | 520(66.4) | <0.0001 | chi2 = 62.953 ; df = 8 | <0.0001 |
|  | 1 | 218(19.7) | 271(17.7) | 78(16.8) | 41(19) | 109(13.9) |  |  |  |
|  | 2-20 | 338(30.5) | 367(23.9) | 96(20.6) | 63(29.2) | 154(19.7) |  |  |  |
| History mixed episodes (lifetime) | no | 842(84) | 1133(81.4) | 308(76.4) | 145(72.5) | 479(69.8) | <0.0001 | chi2 = 61.45 ; df = 4 | <0.0001 |
|  | yes | 160(16) | 259(18.6) | 95(23.6) | 55(27.5) | 207(30.2) |  |  |  |
| Hospitalisation (lifetime) | no | 334(30.8) | 489(32.3) | 91(19.7) | 26(11.8) | 52(6.7) | <0.0001 | chi2 = 231.593 ; df = 4 | <0.0001 |
|  | yes | 750(69.2) | 1027(67.7) | 370(80.3) | 195(88.2) | 726(93.3) |  |  |  |
| Number of hospitalisation (lifetime) |  | 2.013(2.449) | 1.824(2.22) | 2.529(2.61) | 3.222(3.3) | 4.361(3.949) | <0.0001 | F = 120.57 ; df = 4 ; dfres = 4055 | <0.0001 |
| Hospitalisation (last year) | no | 512(63.1) | 808(67.1) | 202(55.8) | 88(54) | 328(49.5) | <0.0001 | chi2 = 63.834 ; df = 4 | <0.0001 |
|  | yes | 300(36.9) | 397(32.9) | 160(44.2) | 75(46) | 335(50.5) |  |  |  |
| Number of hospitalisation (last year) |  | 0.441(0.642) | 0.409(0.667) | 0.669(0.951) | 0.589(0.743) | 0.75(0.943) | <0.0001 | F = 27.111 ; df = 4 ; dfres = 3200 | <0.0001 |
| SA (last year) | no | 632(98.6) | 824(97.7) | 245(95.3) | 131(97) | 410(89.9) | <0.0001 | chi2 = 64.693 ; df = 4 | <0.0001 |
|  | yes | 9(1.4) | 19(2.3) | 12(4.7) | 4(3) | 46(10.1) |  |  |  |
| SI (last year) | no | 453(66.9) | 445(48.5) | 120(42.4) | 81(56.2) | 171(34.3) | <0.0001 | chi2 = 135.741 ; df = 4 | <0.0001 |
|  | yes | 224(33.1) | 472(51.5) | 163(57.6) | 63(43.8) | 327(65.7) |  |  |  |
| MDD (follow 12 month) | no | 506(69.7) | 551(58.7) | 158(56.4) | 97(64.7) | 245(49.1) | <0.0001 | chi2 = 56.697 ; df = 4 | <0.0001 |
|  | yes | 220(30.3) | 388(41.3) | 122(43.6) | 53(35.3) | 254(50.9) |  |  |  |
| Hypomanic episode (follow 12 month) | no | 595(82.6) | 746(80.6) | 234(83) | 120(80.5) | 376(77.2) | 0.16 | chi2 = 6.505 ; df = 4 | 1 |
|  | yes | 125(17.4) | 179(19.4) | 48(17) | 29(19.5) | 111(22.8) |  |  |  |
| Manic episode (follow 12 month) | no | 677(93.9) | 897(96.6) | 273(96.8) | 144(96) | 470(95.9) | 0.08 | chi2 = 8.247 ; df = 4 | 1 |
|  | yes | 44(6.1) | 32(3.4) | 9(3.2) | 6(4) | 20(4.1) |  |  |  |
| Mixed episode (follow 12 month) | no | 686(95.8) | 880(95.2) | 261(92.9) | 140(95.9) | 440(90.5) | 0.0008 | chi2 = 19.044 ; df = 4 | 0.13 |
|  | yes | 30(4.2) | 44(4.8) | 20(7.1) | 6(4.1) | 46(9.5) |  |  |  |
| Mood episode (follow 12 month) | no | 414(57.3) | 477(50.8) | 134(47.3) | 79(52.7) | 203(41.3) | <0.0001 | chi2 = 31.464 ; df = 4 | 0.0004 |
|  | yes | 309(42.7) | 462(49.2) | 149(52.7) | 71(47.3) | 289(58.7) |  |  |  |
| Hospitalisation (follow 12 month) | no | 598(86.5) | 792(88.8) | 218(79.3) | 117(83.6) | 358(74.9) | <0.0001 | chi2 = 52.929 ; df = 4 | <0.0001 |
|  | yes | 93(13.5) | 100(11.2) | 57(20.7) | 23(16.4) | 120(25.1) |  |  |  |
| Smoking status (lifetime) | no | 598(46.2) | 687(43.8) | 195(40.2) | 79(36.7) | 314(37) | <0.0001 | chi2 = 32.197 ; df = 8 | 0.01 |
|  | current | 546(42.2) | 689(43.9) | 241(49.7) | 103(47.9) | 443(52.2) |  |  |  |
|  | past | 151(11.7) | 194(12.4) | 49(10.1) | 33(15.3) | 91(10.7) |  |  |  |
| Alcohol use disorder (lifetime) | no | 897(81.8) | 1160(76) | 335(72.8) | 159(71) | 517(63.3) | <0.0001 | chi2 = 88.36 ; df = 4 | <0.0001 |
|  | yes | 200(18.2) | 366(24) | 125(27.2) | 65(29) | 300(36.7) |  |  |  |
| Cannabis use (lifetime) | no | 930(84.8) | 1198(78.5) | 351(76.3) | 168(75) | 640(78.3) | <0.0001 | chi2 = 26.101 ; df = 4 | 0.005 |
|  | yes | 167(15.2) | 328(21.5) | 109(23.7) | 56(25) | 177(21.7) |  |  |  |
| Cocaine use (lifetime) | no | 1058(96.4) | 1455(95.3) | 438(95.2) | 209(93.3) | 749(91.7) | <0.0001 | chi2 = 24.233 ; df = 4 | 0.01 |
|  | yes | 39(3.6) | 71(4.7) | 22(4.8) | 15(6.7) | 68(8.3) |  |  |  |
| Substance use (lifetime, without alcohol) | no | 898(81.9) | 1146(75.1) | 320(69.6) | 154(68.8) | 555(67.9) | <0.0001 | chi2 = 59.937 ; df = 4 | <0.0001 |
|  | yes | 199(18.1) | 380(24.9) | 140(30.4) | 70(31.2) | 262(32.1) |  |  |  |
| Age at first psychotropic use (treatment) |  | 28.285(10.754) | 28.082(10.344) | 26.23(9.909) | 28.516(10.585) | 25.111(9.748) | <0.0001 | F = 15.551 ; df = 4 ; dfres = 4075 | <0.0001 |
| ***Assessment scales*** |  |  |  |  |  |  |  |  |  |
| ALS score |  | 1.103(0.666) | 1.273(0.636) | 1.472(0.662) | 1.238(0.627) | 1.496(0.657) | <0.0001 | F = 57.458 ; df = 4 ; dfres = 4379 | <0.0001 |
| MARS score |  | 7.088(2.089) | 6.808(1.985) | 6.66(2.092) | 7.136(1.942) | 6.739(2.139) | <0.0001 | F = 6.874 ; df = 4 ; dfres = 4299 | 0.003 |
| BIS 10 score |  | 2.22(0.843) | 2.219(0.854) | 2.232(0.882) | 2.332(0.938) | 2.283(0.96) | 0.19 | F = 1.535 ; df = 4 ; dfres = 4432 | 1 |
| WURS score |  | 29.188(19.31) | 32.265(18.389) | 35.52(19.428) | 32.634(20.608) | 38.415(21.057) | <0.0001 | F = 31.287 ; df = 4 ; dfres = 4383 | <0.0001 |
| CTQ emotional abuse |  | 9.233(4.886) | 9.958(4.735) | 11.372(5.512) | 10.637(5.04) | 12.346(5.997) | <0.0001 | F = 55.127 ; df = 4 ; dfres = 4482 | <0.0001 |
| CTQ emotional abuse (no/yes) | <16 | 1135(87.4) | 1359(85.3) | 388(76.7) | 189(83.6) | 600(69.4) | <0.0001 | chi2 = 139.041 ; df = 4 | <0.0001 |
|  | >= 16 | 163(12.6) | 234(14.7) | 118(23.3) | 37(16.4) | 264(30.6) |  |  |  |
| CTQ physical abuse |  | 6.165(2.496) | 6.342(2.571) | 7.178(3.609) | 6.81(3.125) | 7.275(3.866) | <0.0001 | F = 25.721 ; df = 4 ; dfres = 4486 | <0.0001 |
| CTQ physical abuse (no/yes) | <11 | 1209(93.1) | 1466(91.9) | 440(86.8) | 202(89.4) | 733(84.8) | <0.0001 | chi2 = 52.275 ; df = 4 | <0.0001 |
|  | >= 11 | 90(6.9) | 129(8.1) | 67(13.2) | 24(10.6) | 131(15.2) |  |  |  |
| CTQ sexual abuse |  | 6.082(2.908) | 6.288(3.136) | 6.759(3.699) | 6.533(3.16) | 7.66(4.54) | <0.0001 | F = 30.988 ; df = 4 ; dfres = 4486 | <0.0001 |
| CTQ emotional neglect |  | 11.289(4.796) | 11.897(4.904) | 13.105(5.035) | 12.693(5.213) | 13.747(5.267) | <0.0001 | F = 37.815 ; df = 4 ; dfres = 4479 | <0.0001 |
| CTQ emotional neglect (no/yes) | <16 | 1053(81.2) | 1219(76.5) | 337(66.6) | 167(74.2) | 545(63.2) | <0.0001 | chi2 = 107.83 ; df = 4 | <0.0001 |
|  | >= 16 | 243(18.8) | 375(23.5) | 169(33.4) | 58(25.8) | 318(36.8) |  |  |  |
| CTQ physical neglect |  | 6.922(2.542) | 6.966(2.47) | 7.761(3.077) | 7.626(3.061) | 7.837(3.173) | <0.0001 | F = 24.308 ; df = 4 ; dfres = 4487 | <0.0001 |
| CTQ physical neglect (no/yes) | <14 | 1257(96.9) | 1556(97.4) | 476(93.9) | 213(93.8) | 807(93.4) | <0.0001 | chi2 = 34.552 ; df = 4 | <0.0001 |
|  | >= 14 | 40(3.1) | 41(2.6) | 31(6.1) | 14(6.2) | 57(6.6) |  |  |  |
| CTQ total score |  | 39.703(13.249) | 41.438(12.803) | 46.17(15.574) | 44.338(14.907) | 48.813(16.972) | <0.0001 | F = 64.625 ; df = 4 ; dfres = 4465 | <0.0001 |
| QIDSR score |  | 8.659(5.662) | 10.156(6.111) | 11.41(6.025) | 10.078(6.604) | 12.228(6.187) | <0.0001 | F = 51.05 ; df = 4 ; dfres = 4497 | <0.0001 |
| MADRS score |  | 8.527(8.134) | 11.287(9.352) | 12.144(9.75) | 10.5(9.068) | 13.297(9.768) | <0.0001 | F = 41.014 ; df = 4 ; dfres = 4614 | <0.0001 |
| PSQI score |  | 6.498(3.616) | 7.104(3.616) | 8.24(3.788) | 6.893(3.415) | 8.553(4.054) | <0.0001 | F = 48.729 ; df = 4 ; dfres = 4414 | <0.0001 |
| STAY A score |  | 40.013(14.044) | 43.311(14.957) | 44.988(14.908) | 43.716(15.784) | 47.276(15.205) | <0.0001 | F = 33.245 ; df = 4 ; dfres = 4491 | <0.0001 |
| FAST score |  | 18.45(14.149) | 21.086(14.157) | 23.448(14.478) | 21.849(14.903) | 26.082(15.524) | <0.0001 | F = 37.706 ; df = 4 ; dfres = 4397 | <0.0001 |
| YMRS score |  | 2.243(3.913) | 2.598(3.863) | 2.386(3.438) | 2.223(3.584) | 2.801(3.948) | 0.007 | F = 3.536 ; df = 4 ; dfres = 4601 | 1 |
| ALTMAN score |  | 2.776(3.504) | 2.986(3.688) | 3.182(3.665) | 2.709(3.468) | 3.308(3.761) | 0.008 | F = 3.456 ; df = 4 ; dfres = 4496 | 1 |
| ***Biological markers*** |  |  |  |  |  |  |  |  |  |
| BMI (kg/m²) |  | 26.004(5.21) | 25.421(4.847) | 25.492(5.09) | 25.813(4.7) | 25.931(5.563) | 0.02 | F = 2.957 ; df = 4 ; dfres = 4411 | 1 |
| Abdominal circumference (cm) |  | 92.863(14.648) | 91.031(14.388) | 90.624(14.972) | 92.68(13.199) | 92.419(15.709) | 0.004 | F = 3.887 ; df = 4 ; dfres = 4216 | 0.65 |
| Albumin concentration (g/L) |  | 43.725(3.882) | 43.481(3.904) | 43.467(3.924) | 43.454(3.742) | 43.209(3.904) | 0.1 | F = 1.921 ; df = 4 ; dfres = 3736 | 1 |
| Cholesterol total concentration (g/L) |  | 1.958(0.428) | 1.939(0.437) | 1.98(0.43) | 2.007(0.43) | 1.979(0.445) | 0.1 | F = 1.955 ; df = 4 ; dfres = 3934 | 1 |
| Chol total / chol HDL |  | 3.796(1.4) | 3.689(1.31) | 3.68(1.281) | 4.014(1.39) | 3.739(1.379) | 0.01 | F = 3.171 ; df = 4 ; dfres = 3886 | 1 |
| Cholesterol HDL (g/L) |  | 0.556(0.17) | 0.559(0.16) | 0.575(0.159) | 0.532(0.162) | 0.567(0.164) | 0.02 | F = 2.972 ; df = 4 ; dfres = 3908 | 1 |
| Cholesterol LDL (g/L) |  | 1.164(0.365) | 1.165(0.373) | 1.203(0.389) | 1.234(0.355) | 1.185(0.397) | 0.06 | F = 2.29 ; df = 4 ; dfres = 3704 | 1 |
| Triglycerides (g/L) |  | 1.159(0.676) | 1.107(0.633) | 1.108(0.633) | 1.206(0.645) | 1.148(0.638) | 0.1 | F = 1.924 ; df = 4 ; dfres = 3869 | 1 |
| Urate (umol/L) |  | 304.524(83.745) | 294.437(81.718) | 284.479(79.638) | 307.28(81.523) | 288.865(82.047) | <0.0001 | F = 7.16 ; df = 4 ; dfres = 3658 | 0.002 |
| Bilirubin total (umol/L) |  | 8.425(5.002) | 8.127(4.677) | 7.107(3.823) | 8.185(4.537) | 7.438(4.507) | <0.0001 | F = 9.056 ; df = 4 ; dfres = 3725 | <0.0001 |
| log (Bilirubin total) (umol/L) |  | 2.064(0.49) | 2.038(0.474) | 1.931(0.433) | 2.052(0.46) | 1.949(0.482) | <0.0001 | F = 10.716 ; df = 4 ; dfres = 3725 | <0.0001 |
| TSH (mUI/L) |  | 2.317(1.304) | 2.186(1.297) | 2.148(1.162) | 2.385(1.271) | 2.308(1.38) | 0.02 | F = 2.793 ; df = 4 ; dfres = 3453 | 1 |
| CRP (mg/L) |  | 2.73(2.619) | 2.91(2.515) | 3.21(2.816) | 3.021(2.404) | 3.209(2.907) | 0.001 | F = 4.631 ; df = 4 ; dfres = 3697 | 0.17 |
| log (CRP) (mg/L) |  | 1.1(0.654) | 1.163(0.646) | 1.217(0.679) | 1.208(0.63) | 1.214(0.676) | 0.001 | F = 4.405 ; df = 4 ; dfres = 3697 | 0.26 |
| Leucocytes (G/L) (corrected) |  | 6.888(1.986) | 6.826(2.024) | 6.984(2.092) | 7.045(2.168) | 7.104(2.105) | 0.03 | F = 2.647 ; df = 4 ; dfres = 3938 | 1 |
| Red blood cells (G/L) |  | 4.653(0.466) | 4.62(0.439) | 4.543(0.408) | 4.697(0.459) | 4.506(0.42) | <0.0001 | F = 17.578 ; df = 4 ; dfres = 3899 | <0.0001 |
| Haemoglobin (g/dl) |  | 14.118(1.317) | 14.058(1.257) | 13.781(1.213) | 14.131(1.35) | 13.727(1.213) | <0.0001 | F = 15.432 ; df = 4 ; dfres = 3867 | <0.0001 |
| Haematocrit (Ht) |  | 41.951(3.708) | 41.722(3.533) | 41.089(3.383) | 42.074(3.753) | 41.042(3.478) | <0.0001 | F = 10.203 ; df = 4 ; dfres = 3658 | <0.0001 |
| Neutrophils (G/L) |  | 3.923(1.432) | 3.819(1.404) | 3.985(1.548) | 4.036(1.544) | 4.033(1.49) | 0.01 | F = 3.26 ; df = 4 ; dfres = 3773 | 1 |
| Basophiles (G/L) |  | 0.033(0.025) | 0.032(0.026) | 0.032(0.025) | 0.036(0.028) | 0.035(0.025) | 0.03 | F = 2.595 ; df = 4 ; dfres = 3765 | 1 |
| Eosinophils (G/L) |  | 0.196(0.131) | 0.197(0.136) | 0.193(0.13) | 0.221(0.136) | 0.191(0.128) | 0.1 | F = 1.953 ; df = 4 ; dfres = 3745 | 1 |
| Lymphocytes (G/L) |  | 2.071(0.683) | 2.087(0.68) | 2.115(0.665) | 2.119(0.667) | 2.145(0.667) | 0.19 | F = 1.535 ; df = 4 ; dfres = 3764 | 1 |
| Monocytes (G/L) |  | 0.496(0.16) | 0.499(0.163) | 0.492(0.17) | 0.5(0.161) | 0.492(0.166) | 0.87 | F = 0.305 ; df = 4 ; dfres = 3771 | 1 |
| Mean Corpuscular Volume (fl) |  | 90.474(4.645) | 90.599(4.65) | 90.991(4.758) | 90.229(4.724) | 91.214(4.812) | 0.004 | F = 3.92 ; df = 4 ; dfres = 3812 | 0.61 |
| Mean Corpuscular Haemoglobin (g/100mL) |  | 30.395(1.673) | 30.496(1.672) | 30.49(1.657) | 30.369(1.738) | 30.47(1.788) | 0.68 | F = 0.575 ; df = 4 ; dfres = 3006 | 1 |
| Mean Corpuscular Haemoglobin Concentration (g/100mL) |  | 33.643(0.983) | 33.646(0.974) | 33.528(1.028) | 33.557(1.172) | 33.458(1.043) | 0.002 | F = 4.259 ; df = 4 ; dfres = 2991 | 0.34 |
| Platelets (G/L) |  | 247.737(60.34) | 247.11(57.649) | 255.371(62.943) | 254.554(61.243) | 259.059(64.269) | <0.0001 | F = 6.21 ; df = 4 ; dfres = 3817 | 0.01 |
| Lymphocytes (%) |  | 31.423(9.336) | 31.949(8.56) | 31.926(8.956) | 31.59(9.119) | 31.961(9.333) | 0.62 | F = 0.663 ; df = 4 ; dfres = 3764 | 1 |
| Neutrophils (%) |  | 56.997(9.976) | 56.32(9.824) | 56.91(9.689) | 56.743(9.558) | 57.001(9.735) | 0.43 | F = 0.965 ; df = 4 ; dfres = 3773 | 1 |
| Monocytes (%) |  | 7.547(2.372) | 7.656(2.178) | 7.363(2.344) | 7.41(2.015) | 7.26(2.088) | 0.002 | F = 4.343 ; df = 4 ; dfres = 3771 | 0.29 |
| Mono/HDL |  | 1.008(0.606) | 0.982(0.491) | 0.939(0.455) | 1.108(1.227) | 0.963(0.504) | 0.01 | F = 3.325 ; df = 4 ; dfres = 3626 | 1 |
| Log (Mono/HDL) |  | -0.125(0.516) | -0.136(0.5) | -0.178(0.493) | -0.077(0.54) | -0.167(0.524) | 0.09 | F = 2.016 ; df = 4 ; dfres = 3626 | 1 |
| NLR |  | 2.096(1.088) | 2.007(1.052) | 2.041(1.032) | 2.065(1.073) | 2.039(0.969) | 0.36 | F = 1.096 ; df = 4 ; dfres = 3755 | 1 |
| log (NLR) |  | 0.684(0.449) | 0.648(0.432) | 0.666(0.429) | 0.675(0.433) | 0.667(0.431) | 0.39 | F = 1.025 ; df = 4 ; dfres = 3755 | 1 |
| MLR |  | 0.258(0.107) | 0.255(0.095) | 0.245(0.092) | 0.249(0.087) | 0.242(0.089) | 0.004 | F = 3.897 ; df = 4 ; dfres = 3757 | 0.64 |
| log (MLR) |  | -1.435(0.427) | -1.439(0.397) | -1.476(0.389) | -1.455(0.393) | -1.493(0.412) | 0.02 | F = 3.004 ; df = 4 ; dfres = 3757 | 1 |
| PLR |  | 131.503(50.513) | 129.498(49.514) | 130.697(47.773) | 131.323(50.028) | 131.544(51.052) | 0.86 | F = 0.322 ; df = 4 ; dfres = 3732 | 1 |
| log (PLR) |  | 4.811(0.37) | 4.799(0.358) | 4.809(0.363) | 4.808(0.375) | 4.812(0.366) | 0.93 | F = 0.214 ; df = 4 ; dfres = 3732 | 1 |
| ***Treatment*** |  |  |  |  |  |  |  |  |  |
| Antidepressant (n06a) | no | 981(68.7) | 964(57.9) | 293(56.1) | 134(56.3) | 457(50.6) | <0.0001 | chi2 = 85.413 ; df = 4 | <0.0001 |
|  | yes | 446(31.3) | 700(42.1) | 229(43.9) | 104(43.7) | 446(49.4) |  |  |  |
| Anticonvulsant (n03a) | no | 826(57.9) | 801(48.1) | 248(47.5) | 118(49.6) | 404(44.7) | <0.0001 | chi2 = 48.76 ; df = 4 | <0.0001 |
|  | yes | 601(42.1) | 863(51.9) | 274(52.5) | 120(50.4) | 499(55.3) |  |  |  |
| Antipsychotic (n05a except n05an) | no | 831(58.2) | 925(55.6) | 266(51) | 133(55.9) | 445(49.3) | 0.0003 | chi2 = 21.511 ; df = 4 | 0.044 |
|  | yes | 596(41.8) | 739(44.4) | 256(49) | 105(44.1) | 458(50.7) |  |  |  |
| Lithium (n05an) | no | 962(67.4) | 1058(63.6) | 345(66.1) | 137(57.6) | 571(63.2) | 0.02 | chi2 = 12.098 ; df = 4 | 1 |
|  | yes | 465(32.6) | 606(36.4) | 177(33.9) | 101(42.4) | 332(36.8) |  |  |  |
| Anxiolytic (n05b) | no | 1154(80.9) | 1292(77.6) | 382(73.2) | 182(76.5) | 597(66.1) | <0.0001 | chi2 = 71.412 ; df = 4 | <0.0001 |
|  | yes | 273(19.1) | 372(22.4) | 140(26.8) | 56(23.5) | 306(33.9) |  |  |  |
| Hypnotic (n05c) | no | 1313(92) | 1481(89) | 457(87.5) | 206(86.6) | 754(83.5) | <0.0001 | chi2 = 41.451 ; df = 4 | <0.0001 |
|  | yes | 114(8) | 183(11) | 65(12.5) | 32(13.4) | 149(16.5) |  |  |  |
| Treatment clusters 4 classes | polytherapy without lithium | 325(22.8) | 480(28.8) | 166(31.8) | 59(24.8) | 284(31.5) | <0.0001 | chi2 = 82.088 ; df = 12 | <0.0001 |
|  | monotherapy without lithium | 637(44.6) | 578(34.7) | 179(34.3) | 78(32.8) | 287(31.8) |  |  |  |
|  | bitherapy with lithium | 236(16.5) | 294(17.7) | 76(14.6) | 46(19.3) | 121(13.4) |  |  |  |
|  | polytherapy with lithium | 229(16) | 312(18.8) | 101(19.3) | 55(23.1) | 211(23.4) |  |  |  |
| ***Cognitive battery*** |  |  |  |  |  |  |  |  |  |
| Digit symbol coding (std) |  | 65.176(16.21) | 66.596(15.628) | 66.709(14.326) | 63.979(15.32) | 64.381(14.9) | 0.006 | F = 3.633 ; df = 4 ; dfres = 3622 | 1 |
| WAIS symb total score (std) |  | 10.019(2.894) | 10.122(2.927) | 10.168(2.812) | 9.912(2.864) | 9.951(2.894) | 0.59 | F = 0.706 ; df = 4 ; dfres = 3620 | 1 |
| Digit span (WAIS) |  | 9.617(2.92) | 9.908(2.733) | 9.463(2.721) | 9.264(2.793) | 9.204(2.699) | <0.0001 | F = 8.381 ; df = 4 ; dfres = 3617 | 0.0002 |
| Continuous Performance Test (omission) |  | 63.984(29.761) | 58.132(28.95) | 64.096(28.349) | 58.787(31.054) | 65.149(28.228) | <0.0001 | F = 6.288 ; df = 4 ; dfres = 2243 | 0.009 |
| Continuous Performance Test (commission) |  | 51.561(11.44) | 50.795(10.502) | 52.199(11.825) | 52.35(11.648) | 53.094(11.533) | 0.01 | F = 3.211 ; df = 4 ; dfres = 2229 | 1 |
| STROOP note Z |  | -0.051(1.085) | 0.014(1.054) | -0.11(1.065) | -0.121(1.068) | -0.12(1.038) | 0.045 | F = 2.433 ; df = 4 ; dfres = 3565 | 1 |
| CVLT score A (std age) |  | -0.287(1.313) | -0.112(1.329) | -0.186(1.365) | -0.257(1.26) | -0.184(1.305) | 0.03 | F = 2.595 ; df = 4 ; dfres = 3559 | 1 |
| CVLT (long delay free recall) |  | -0.498(1.375) | -0.287(1.235) | -0.234(1.19) | -0.443(1.217) | -0.358(1.284) | 0.042 | F = 2.482 ; df = 4 ; dfres = 1730 | 1 |
| TMTA time (std) |  | 0.113(0.827) | 0.118(0.805) | 0.147(0.838) | 0.021(0.884) | 0.073(0.847) | 0.35 | F = 1.104 ; df = 4 ; dfres = 3568 | 1 |
| TMTB time (std) |  | -0.147(1.183) | -0.104(1.046) | -0.134(1.155) | -0.138(1.17) | -0.193(1.149) | 0.59 | F = 0.698 ; df = 4 ; dfres = 3503 | 1 |
| Mean TMTA TMTB |  | -0.01(0.87) | 0.008(0.782) | 0.013(0.849) | -0.059(0.884) | -0.055(0.868) | 0.5 | F = 0.835 ; df = 4 ; dfres = 3485 | 1 |
| Verbal fluency nb words P (std) |  | -0.002(1.129) | 0.022(1.129) | -0.056(1.123) | -0.071(1.212) | -0.027(1.116) | 0.66 | F = 0.607 ; df = 4 ; dfres = 3584 | 1 |
| Verbal fluency nb words anim (std) |  | -0.291(1.053) | -0.219(1.046) | -0.26(1.093) | -0.323(1.074) | -0.38(1.039) | 0.03 | F = 2.72 ; df = 4 ; dfres = 3574 | 1 |
| Mean Verbal fluency |  | -0.148(0.942) | -0.099(0.938) | -0.16(0.965) | -0.197(0.964) | -0.2(0.931) | 0.19 | F = 1.532 ; df = 4 ; dfres = 3573 | 1 |
| Spatial span forward |  | -0.059(0.897) | 0.002(0.883) | -0.169(0.878) | -0.138(0.921) | -0.25(0.887) | <0.0001 | F = 9.041 ; df = 4 ; dfres = 3197 | <0.0001 |
| Spatial span backward |  | -0.158(0.861) | -0.034(0.876) | -0.243(0.783) | -0.162(0.826) | -0.316(0.901) | <0.0001 | F = 11.753 ; df = 4 ; dfres = 3197 | <0.0001 |
| Mean spatial span |  | -0.109(0.751) | -0.016(0.75) | -0.206(0.7) | -0.15(0.723) | -0.283(0.77) | <0.0001 | F = 13.99 ; df = 4 ; dfres = 3197 | <0.0001 |
| Vocabulary (WAIS) |  | 11.414(3.185) | 11.632(3.133) | 11.378(3.096) | 11.366(3.463) | 11.238(3.149) | 0.22 | F = 1.432 ; df = 4 ; dfres = 2587 | 1 |
| Matrix (WAIS) |  | 9.768(2.717) | 10.058(2.59) | 9.382(2.696) | 9.614(2.608) | 9.611(2.701) | <0.0001 | F = 6.606 ; df = 4 ; dfres = 3526 | 0.005 |
| Mean Vocab matrix (WAIS) |  | 10.605(2.498) | 10.838(2.348) | 10.367(2.356) | 10.473(2.625) | 10.474(2.429) | 0.01 | F = 3.267 ; df = 4 ; dfres = 2557 | 1 |
