# Supplementary material for: Staging of suicidality in bipolar disorder: Findings from the FACE-BD cohort (FondaMental Advanced Centers of Expertise for Bipolar Disorders)
Source: Eur Psychiatry. 2025 Jul 22;68(1):e117. doi: 10.1192/j.eurpsy.2025.10068 (PMC12438995; doi:10.1192/j.eurpsy.2025.10068)
Supplement: Auxilia et al. supplementary material 2 — Auxilia et al. supplementary material [file S0924933825100680sup002.docx]

**Table 3.** Results of ANOVAs testing for between stages differences for each variable belonging to the biology set.

|  | Sum Sq | Df | F value | Pr(>F) |
| --- | --- | --- | --- | --- |
| Albumine (g/L) | 3.046 | 4 | 0.868 | 0.48 |
| Cholesterol total (g/L) | 0.708 | 4 | 0.197 | 0.94 |
| Cholesterol HDL (g/L) | 0.57 | 4 | 0.192 | 0.94 |
| Triglycerides (g/L) | 0.761 | 4 | 0.245 | 0.91 |
| Urate (umol/L) | 1.461 | 4 | 0.587 | 0.67 |
| log (Bilirubin total) (umol/L) | 16.618 | 4 | 4.467 | *0.001* |
| TSH (mUI/L) | 6.914 | 4 | 1.746 | 0.14 |
| Haemoglobin (g/dL) | 6.076 | 4 | 2.66 | *0.03* |
| Mean Corpuscular Haemoglobin (g/100mL) | 7.731 | 4 | 2.127 | 0.08 |
| Mean Corpuscular Haemoglobin Concentration (g/100mL) | 4.12 | 4 | 1.101 | 0.35 |

*Model adjusted for sex, age, education, AUD, SUD, treatment, smoking status and BMI.*
